# Evaluation of Work Satisfaction, Stress, and Burnout Among US Internal Medicine Physicians and Trainees

**DOI:** 10.1001/jamanetworkopen.2020.18758

**Published:** 2020-10-14

**Authors:** Mark Linzer, Cynthia D. Smith, Susan Hingle, Sara Poplau, Richard Miranda, Rebecca Freese, Kerri Palamara

**Affiliations:** 1 Department of Medicine, Hennepin Healthcare, Minneapolis, Minnesota; 2 Medical Education Division, American College of Physicians, Philadelphia, Pennsylvania; 3 Department of Medicine, Southern Illinois University, Springfield; 4 Hennepin Healthcare Research Institute, Minneapolis, Minnesota; 5 Department of Medicine, Division of Graduate Medical Education, SCL Health, Saint Joseph Hospital, Denver, Colorado; 6 Clinical and Translational Science Institute, Biostatistical Design and Analysis Center, University of Minnesota, Minneapolis; 7 Center for Physician Well-being, Massachusetts General Hospital, Boston; 8 Department of Medicine, Harvard Medical School, Boston, Massachusetts; 9 Department of Medicine, Perelman School of Medicine, University of Pennsylvania, Philadelphia

## Abstract

This study uses the Mini Z 2.0 survey to assess burnout among male and female members of the American College of Physicians who are internists and internal medicine trainees.

## Introduction

The clinician burnout epidemic has prompted calls for action by many national organizations.^1,2^ Few baseline data are available on the state of burnout among internal medicine physicians and trainees.^3^ Beginning in 2015, we developed a Well-being Champion (WC) program through the American College of Physicians (ACP) to train leaders to support well-being and measure change throughout the ACP membership worldwide. Herein we describe the survey responses from 1305 internists and internal medicine trainees who participated in the program across 18 ACP chapters and identify potential contributors to burnout as well as sex-based differences in burnout.

## Methods

This study was approved by the Partners Healthcare Institutional Review Board, which waived the requirement for informed consent because only deidentified data were used. A well-being curriculum was delivered (in 2018 and 2019) to approximately 150 ACP chapter-designated WC programs. To understand well-being among chapter members, some WC programs asked members to complete the Mini Z worklife survey. Some WC programs included residents, fellows, and students among those surveyed, whereas others did not. The Mini Z survey measures satisfaction, stress, and burnout and their risk factors, and it is validated against the Maslach Burnout Inventory.^4^ The most recent version of the Mini Z survey (2.0) (Figure) aligns positive scores for the 10 items with a possible summary score of 50. Two 5-item subscales have total scores of 25. A joyful workplace is defined by a summary score of 40 or higher; a supportive work environment is represented by a subscale score of 20 or higher (score range, 5-25) , and a reasonable work pace and stress level associated with electronic medical record (EMR) use is represented by a subscale score of 20 or higher. Data from 1305 Mini Z surveys were summarized using counts and frequencies with predetermined cutoffs.^5^ Multiple logistic regression models were used to assess risk factors of burnout and satisfaction. Risk factors of burnout included stress, work control, atmosphere (chaos), documentation time pressure, teamwork, values alignment, EMR work at home, and EMR frustration; these items were transformed from 5-point Likert scale responses to binary variables by grouping positive responses (eg, strongly agree and agree) and neutral and negative responses (eg, neither agree nor disagree, disagree, and strongly disagree). Sex-based differences in summary scores, burnout, satisfaction, and all of the previously mentioned risk factors for burnout were tested in separate, single logistic regression models. The level of statistical significance was *P* < .05.

**Figure.**
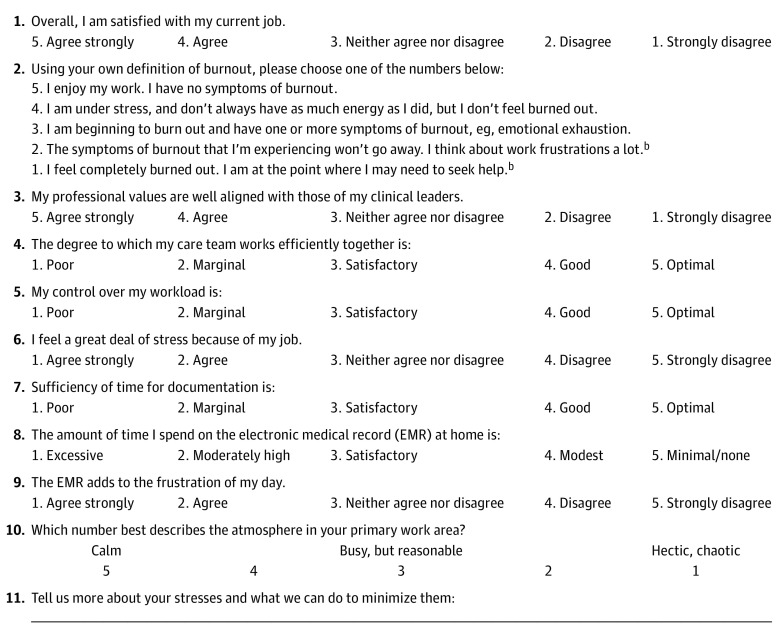
Mini Z 2.0 Survey^a^ ^a^The Mini Z was developed by Mark Linzer, MD and a team at Hennepin Healthcare, Minneapolis, Minnesota. The Mini Z survey tool can be used for research, program evaluation and education capacities without restriction. Permission for commercial or revenue-generating applications of the Mini Z must be obtained from Mark Linzer, MD, or the Hennepin Healthcare Institute for Professional Worklife prior to use (www.professionalworklife.com). ^b^If you select option 1 or 2, please consider seeking assistance; call your insurance provider or employee assistance plan.

## Results

Response rates in the 8 chapters and 2 cohorts of WC programs for whom sampling data were available (n = 11 625) ranged from 2% to 76% (median 9.5%). Among 1270 respondents who indicated their sex, 665 were men (52.4%) and 605 were women (47.6%); 680 respondents (52.1%%) reported symptoms of burnout.

For the single logistic regression models, the reference group was male. Although 938 of 1305 respondents (71.9) reported career satisfaction, the burnout level (52.1%) was high in this sample of ACP members. One-third of participants (n = 419) reported poor or marginal work control, and approximately one-half (n = 673) reported time pressure associated with EMR documentation (Table). In the regressions, burnout was associated with lack of work control (OR, 2.32 [95% CI, 1.66-3.26]; *P* < .001) and documentation time pressure (OR, 1.64 [95% CI, 1.20-2.24]; *P* = .002). Job satisfaction was associated with professional values alignment with those of clinical leaders (OR, 4.24 [95% CI, 3.05-5.81]; *P* < .001) and efficient teamwork (satisfactory to optimal) (OR, 2.47 [95% CI, 1.59-3.87]; *P* < .001). The odds of burnout among women were 56% higher compared with men (Table), and women had 61% lower odds of having a joyous workplace, 39% lower odds of having supportive work environments, and 61% lower odds of having a manageable work pace and manageable EMR-related stress.

**Table.** Overall and Sex-Specific Scores on Satisfaction, Stress, and Burnout and Risk Factors for Burnout Among Internists and Trainees Enrolled in a Well-being Champion Program

Survey item or score (response)	Overall	Female^a^	Male^a^	OR (95% CI)^b^	*P* value
Participants, No. (%)	1305 (100)	605 (47.6)	665 (52.4)	NA	NA
Satisfaction with current job (agree or strongly agree)	938 (71.9)	427 (70.6)	492 (74.0)	0.84 (0.66-1.08)	.18
Burnout symptoms (present to severe)	680 (52.1)	351 (58.0)	312 (46.9)	1.56 (1.25-1.95)	<.001
Values aligned with those of clinical leaders (agree or strongly agree)	816 (62.5)	363 (60.0)	438 (65.9)	0.78 (0.62-0.98)	.03
My care team works efficiently together (satisfactory to optimal)	1128 (86.4)	522 (86.3)	581 (87.4)	0.91 (0.66-1.26)	.57
Personal control over workload (Poor or minimal)	419 (32.1)	206 (34.0)	196 (29.5)	0.81 (0.64-1.03)	.08
Feeling a great deal of stress (agree or strongly agree)	730 (55.9)	376 (62.1)	334 (50.2)	1.63 (1.30-20.4)	<.001
Sufficient time for documentation(poor, marginal)	673 (51.6)	315 (52.1)	335 (50.4)	1.07 (0.86-1.33)	.55
Time spent on EMR at home (moderately high to excessive)	552 (42.3)	268 (44.3)	263 (39.5)	1.22 (0.97-1.52)	.09
EMR adds frustration to the day (agree or strongly agree)	850 (65.1)	383 (63.3)	443 (66.6)	0.86 (0.69-1.09)	.22
Work atmosphere (chaotic or tending toward chaotic)	390 (29.9)	191 (31.6)	188 (28.3)	1.17 (0.92-1.49)	.20
Summary score ≥40 (joyous workplace)^c^	151 (11.6)	42 (6.9)	107 (16.1)	0.39 (0.26-0.56)	<.001
Subscale 1 score ≥20 (supportive workplace)^d^	466 (35.7)	182 (30.1)	275 (41.4)	0.61 (0.48-0.77)	<.001
Subscale 2 score ≥ 20 (manageable work pace and EMR stress)^e^	117 (9.0)	32 (5.3)	83 (12.5)	0.39 (0.25-0.59)	<.001

Abbreviations: EMR, electronic medical record; NA, not applicable; OR, odds ratio.

^a^ Of 1305 respondents, 35 chose not to indicate their sex and are not included in this table.

^b^ All ORs from single logistic regression models are for women compared with men.

^c^ Summary score range 10 to 50. Mean (SD) score: 30.9 (7.4).

^d^ Subscale 1 (including items 1-5) score range 5 to 25. Mean (SD) score: 17.5 (4.1).

^e^ Subscale 2 (including items 6-10) score range 5 to 25. Mean (SD) score: 13.4 (4.1).

## Discussion

Although most of the surveyed ACP members reported career satisfaction (71.9%), burnout levels were high. Risk factors of burnout included documentation time pressure and lack of work control, whereas satisfaction was associated with alignment of professional values with those of the respondents’ clinical leaders and efficient teamwork. As in previous studies,^6^ female clinicians had higher odds of burnout than male clinicians, and were less likely to describe supportive environments or manageable work conditions. This study is limited by the absence of demographic data other than sex and the need for additional validation of the Mini Z 2.0 survey. Although the study is also limited by nonrandom sampling, data from this cohort of ACP members may still be generalizable to other populations for assessment of sex-based differences in potential associations between work conditions and burnout.
